# PP77 Cost-Effectiveness Of The Dengue Vaccine (TAK-003) In Brazil

**DOI:** 10.1017/S0266462324002460

**Published:** 2025-01-07

**Authors:** Ricardo Fernandes, Márcia Gisele da Costa, Bernardo Tura, Bruno Barros, Marisa Santos

## Abstract

**Introduction:**

Dengue is an arbovirus that causes an acute febrile illness transmitted by the Aedes aegypti mosquito. The TAK-003 vaccine produced with Vero cells using recombinant DNA technology from the live and attenuated virus was approved by the Brazilian regulatory agency for preventing dengue infection, regardless of previous infection.

**Methods:**

A cost-effectiveness assessment comparing vaccinating specific age groups versus not vaccinating was conducted through a microsimulation model that tracked all epidemic rates over 20 years. Transitions between five health states (susceptible, asymptomatic, ambulatory, hospitalized, and death) were considered. Direct costs included vaccine costs and dengue healthcare (inpatient and outpatient). Quality-adjusted life years (QALY) were the primary outcome. Deterministic and probabilistic analyses explored key model uncertainties.

**Results:**

At a price of USD34.00 per dose, the average incremental cost-effectiveness ratio (ICER) was USD11,724.40/QALY. In the probabilistic analysis, no simulation was below the threshold of USD8,000.00/QALY. In an alternative scenario, in which the price of the vaccine would not be subject to taxes in the case of direct import, the average ICER was USD10,378,09/QALY, with one percent of the simulations below the standard cost-effectiveness threshold.

**Conclusions:**

At both price scenarios, dengue vaccine did not prove to be a cost-effective technology to be funded for patients between four and 60 years old. Sensitivity analyses showed that vaccine price is the most sensitive variable. This study can support price negotiations to improve access to the vaccine.

